# Cytoskeletal actin patterns shape mast cell activation

**DOI:** 10.1038/s42003-019-0322-9

**Published:** 2019-03-07

**Authors:** Huw Colin-York, Dong Li, Kseniya Korobchevskaya, Veronica T. Chang, Eric Betzig, Christian Eggeling, Marco Fritzsche

**Affiliations:** 1 MRC Human Immunology Unit, Weatherall Institute of Molecular Medicine, University of Oxford, Headley Way, Oxford, OX3 9DS UK; 2 Howard Hughes Medical Institute, Janelia Research Campus, 19700 Helix Drive, Ashburn, VA 20147 USA; 3 National Laboratory of Biomacromolecules, Institute of Biophysics, Chinese Academy of Sciences, Beijing, 100101 China; 4 Kennedy Institute for Rheumatology, University of Oxford, Roosevelt Drive, Oxford, OX3 7LF UK; 5 MRC Laboratory of Molecular Biology, University of Cambridge, Cambridge, CB2 0QH UK

## Abstract

Activation of immune cells relies on a dynamic actin cytoskeleton. Despite detailed knowledge of molecular actin assembly, the exact processes governing actin organization during activation remain elusive. Using advanced microscopy, we here show that Rat Basophilic Leukemia (RBL) cells, a model mast cell line, employ an orchestrated series of reorganization events within the cortical actin network during activation. In response to IgE antigen-stimulation of FCε receptors (FCεR) at the RBL cell surface, we observed symmetry breaking of the F-actin network and subsequent rapid disassembly of the actin cortex. This was followed by a reassembly process that may be driven by the coordinated transformation of distinct nanoscale F-actin architectures, reminiscent of self-organizing actin patterns. Actin patterns co-localized with zones of Arp2/3 nucleation, while network reassembly was accompanied by myosin-II activity. Strikingly, cortical actin disassembly coincided with zones of granule secretion, suggesting that cytoskeletal actin patterns contribute to orchestrate RBL cell activation.

## Abstract (web summary)

Huw Colin-York et al. use advanced microscopy techniques to show that the cortical actin network within a model mast cell line undergoes a series of reorganizational events at the basal interface during activation. They find that actin patterns co-localize with zones of Arp2/3 nucleation and myosin-II activity accompanies network reassembly.

## Introduction

Activation of immune cells is partly governed by the biophysics of the cortical actin cytoskeleton. The principles by which the cortical actin cytoskeleton modulates processes essential to the immune response such as receptor-antigen binding and granule exocytosis remain to a great part elusive^1–3^. Two fundamentally different mechanisms exist to generate macromolecular structures in living cells: self-assembly and self-organization^4,5^. Self-assembly is the physical association of molecules into an equilibrium structure with no energy dissipation or external intervention, purely driven by the tendency of systems to minimize their free energy in accordance with the second law of thermodynamics^6,7^. Self-assembly commonly depends on a template of cellular programs encoded and decoded by signaling and transcription^8^. Prominent examples of self-assembly are protein folding or the phase separation of lipids and proteins due to macromolecular interactions such as lipid packing. Phase separation of lipids occurs if the interaction energies dominate the entropy contribution^9,10^. Self-organization, on the other hand, requires the collective action of interacting molecules far from thermodynamic equilibrium driven by the constant input of energy into a steady-state structure, characteristic of reaction–diffusion systems^4,11^. In practice, cellular order results from both a combination of complex deterministic interactions (self-assembly) brought about by specific signaling events and from dynamic interactions between molecules that require energy dissipation (self-organization)^12,13^.

The actin cortex fulfills all criteria of self-organization^5,14^. It continuously consumes energy to maintain its steady state, and changes in the local or global biophysical parameters, such as mechanical stress, can induce spontaneous symmetry breaking^15,16^. Symmetry breaking is a phenomenon in which small fluctuations acting on a system crossing a critical point decide the system's steady state^5,15^. Such symmetry breaking events give rise to instabilities within the network which can rapidly form new order such as distinct filamentous actin (F-actin) architectures^17^. Employing self-organized principles enables cells to rapidly transform their F-actin networks, for example, from isotropic random networks into ordered F-actin networks structured by actin patterns such as actin vortices and asters^18^.

Despite the overwhelming evidence of self-organizing actin patterns in vitro^14,19,20^ and predictions of such patterns in living cells^21^, only recently have we been able to directly demonstrate how self-organizing patterns of the actin cortex in the form of actin vortices and asters govern cortex homeostasis and function in living cells^17^. Using advanced optical microscopy with extended spatial and temporal resolution, we showed in live cervical HeLa cells how self-organizing actin patterns were dynamically formed, nucleated and maintained by the Arp2/3 complex, and underwent a series of transformations from actin vortices to asters in order to produce new F-actin networks and to facilitate cell adherence^17^. Importantly, the actin patterns formed at differently sized regions all over the cell volume, suggesting that cytoskeletal actin patterns robustly and dynamically adjusted their organization to environmental cues and thus to the needs of the cell, an essential property of self-organization. While no self-organizing actin patterns have so far been reported in immune cells, efficient actin network reorganization is known to be essential for effective immune responses^22,23^. Actin networks in lymphocytes have not provided any evidence of the presence of self-organization suggesting self-assembly to be dominant^24–26^.

Historically, advances in understanding the principles underlying key events in actin organization have been driven by innovations in microscopy that provided greater spatial and temporal resolution coupled to appropriate immuno-chemical, genetic and biochemical tools. However, until recently, state-of-the-art microscopy has not been sufficiently informative to dissect the underlying real-time spatio-temporal dynamics of the actin cytoskeleton due to missing spatial and temporal resolution.

Here, we investigate cortical F-actin dynamics during the activation of rat basophilic leukemia (RBL) cells, a model system for FCε receptor (FCεR)-mediated immune cell activation. Using high-resolution optical extended total-internal-reflection-fluorescence coupled with structured-illumination microscopy (eTIRF-SIM), as well as super-resolution stimulated emission depletion (STED) microscopy, we monitored F-actin dynamics at extended spatial and temporal resolution in living RBL cells. We found a new process by which cytoskeletal actin patterns contribute to the spatio-temporal organization of RBL cell activation. Specifically, symmetry breaking resulted in F-actin network disassembly, accompanied by coordinated transformations of distinct nanoscale F-actin architectures, reminiscent of self-organizing actin patterns. In addition, cortical disassembly coincided with zones of granule secretion, indicating that tuning cytoskeletal actin dynamics might thus be a process by which RBL cells can efficiently coordinate their activation.

## Results

### Actin undergoes global rearrangements during activation

We chose the well-established system of RBL-2H3 cells expressing FCεR as a model system of mast and basophil cells and studied their activation through clustering of FCεRs by IgE-antibody binding. This was achieved by exposing the RBL cells to microscope coverglass coated with TNP-BSA (2,4,6-Trinitrophenyl hapten conjugated Bovine Serum Albumin) crosslinked IgE (see Methods; Supplementary Figure 1A)^27–29^. Modified glass surfaces are widely used to study the earliest stages of immune cell activation^30–32^ and they allowed us to straightforwardly compare results for contact formation with and without antigens.

**Fig. 1:**
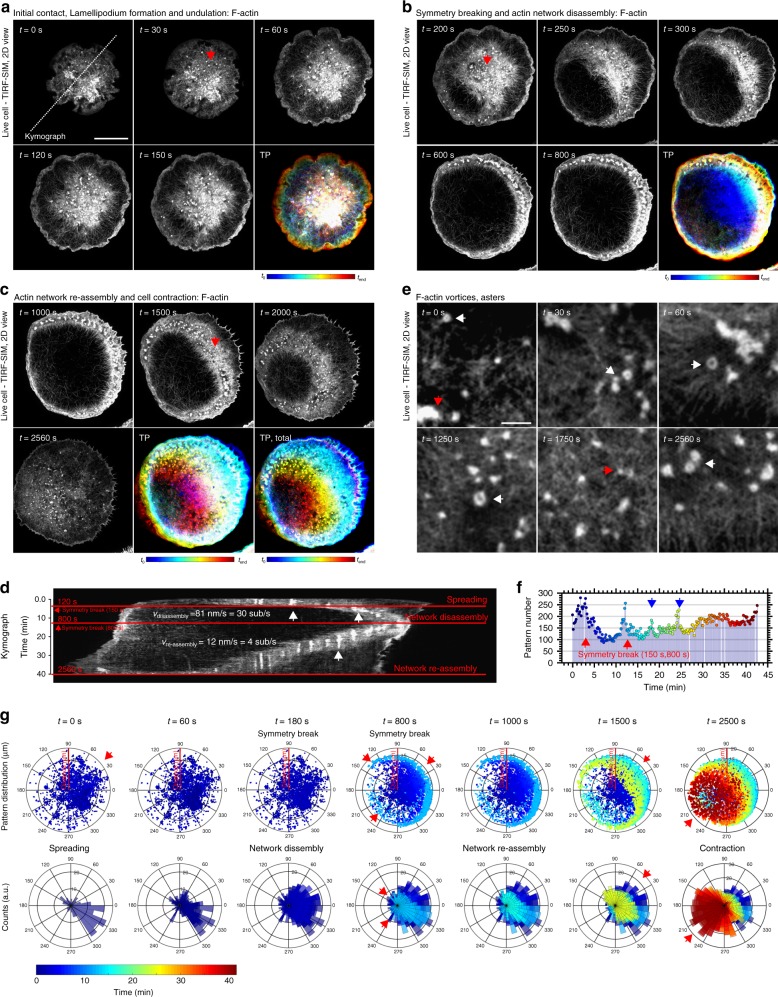
Spatio-temporal actin cytoskeleton reorganization during rat basophilic leukemia (RBL) cell activation. Representative extended total-internal-reflection-fluorescence coupled with structured-illumination microscopy (eTIRF-SIM) experiment of F-actin (Lifeact-citrine) at the basal membrane of RBL cells at different times *t* after contact formation with the activating microscope coverglass. **a**–**c** Images outlining the different stages of initial contact formation and lamellipodium formation and undulation (**a**, *t* = 0–150 s), symmetry breaking and central F-actin network disassembly (**b**, *t* = 200–800 s) and F-actin network reassembly (**c**, *t* = 1000–2560 s). The overall actin reorganization is highlighted by the respective temporal projections (TPs, lower right panels, total *t* = 0–2560 s) with cold colors (early times) transforming into warm colors (late times). Red arrows point at representative actin patterns. Scale bar: 10 μm. **d** Temporal kymograph along the dashed line marked in **a** (intensity at the same spatial *x*-positions along the line over time with time as *y*-axis starting from top), and with red horizontal lines indicating the different stages, spreading at *t* < 120 s, symmetry break at 150 s, central network disassembly until 800 s, at the time point of the next symmetry break, and finally central network reassembly until 2560 s. Tracking of the positions of the edges over space and time determines the velocities *v*_disassembly_ and *v*_reassembly_ of the disassembling and re-assembling actin waves. **e** Zoom-in into different parts of **a**–**c** at times *t* as marked, highlighting the appearance of actin patterns such as asters (red arrows) and vortices (white arrows). Scale bar: 1 μm. **f** Total number of patterns identified at different times *t* from recordings on 35 cells, indicating peaks at the time-points of symmetry breaking (red arrows, *t* = 150 s and 800 s) and at later time-points (blue arrows). **g** Localization of individual actin patters at different time-points *t* as taken from **a**–**c**: (upper panels) scatter-plot of spatial positions within the circular cell interface, and (lower panels) directional histograms with the number of patterns identified within a certain segment of the circular cell interface displayed by the lengths of the respective segmental column bars. Cold blue colors indicate early time-points and warm red colors late time-points. The patterns cumulated at the leading edge and actively followed the propagating F-actin

We performed eTIRF-SIM^25,33^ as well as three-dimensional (3D) STED microscopy^34^ to monitor the sequence of cytoskeletal network rearrangements during RBL activation. To this end, we labeled F-actin with Lifeact-citrine in live RBL cells. eTIRF-SIM enabled us to follow the processes at the contact interface with high spatial (~90 nm laterally and down to ~100 nm axially) and temporal resolution (up to 10 frames per second) with an image acquisition every 30 s from the first contact until 1 h after initial contact. 3D-STED microscopy allowed the three-dimensional spatial analysis of the cytoskeletal network with enhanced lateral (here ~60 nm) and improved axial (here ~300 nm) resolution. We identified four stages of F-actin rearrangements at different time-points between 0 and 50 min after initial contact formation with the activating glass dishes (Fig. 1a–c for eTIRF-SIM and Supplementary Figure 1B-D for STED).

Within the first 60 s, the RBL cells produced a circular contact with an interface comprised of a dense network of F-actin (42 of 42 cells), which centripetally spread until the contact interface reached its maximum extension (35 of 42 cells) (Fig. 1a, upper panels *t* = 0–60 s, and Supplementary Figure 1B *t* = 0 s).

At about 60 s, the RBL cells formed a lamellipodium at the leading edge of the symmetric contact interface and a coarse central F-actin network with bright cluster-like actin features in the central area. When the spreading process slowed down, the lamellipodium produced continuous undulations at the outer cell periphery (35 of 35 spreading cells; Fig. 1a, lower panels *t* = 120 s and 150 s, and Supplementary Movie 1). Temporal projections (TPs) of the spreading process further visualize the dynamics of the F-actin network (Fig. 1a, TP) and the cellular periphery (Supplementary Figure 2), where blue to red colors report on early to late time-points. A closer visual inspection of the 3D-STED images at this stage revealed that the lamellipodium structure consisted of primarily a dense network of short and a few long length F-actins, which could not be further resolved, suggesting that the lamellipodial mesh-size was smaller than the spatial resolution of 60 nm. At the rear of the lamellipodium, the network extended to a larger ring-like F-actin network that interconnected to a second coarser network at the interface center with predominantly longer F-actins (25 of 25 spreading cells; Supplementary Figure 1B, *t* = 60 s).

At around 200 s after contact formation, the symmetry of the F-actin network spontaneously broke, and the central F-actin network transformed into a non-symmetric wave-like F-actin structure, resulting in complete disassembly of F-actin in the center of the contact interface (32 of the 35 spreading cells). Figure 1b shows representative images of the disassembly process, which, in this case, traveled from the lower left of the contact interface to the upper right (see also Supplementary Movie 1). Complete F-actin disassembly was reached at around 500 s. The propagation of the symmetry breaking event over time is further illustrated by the temporal projection turning red at full disassembly (Fig. 1b, TP).

After full opening of the cortical F-actin network, the wave-like structure reversed its propagation direction, transforming into an F-actin reassembly process, resulting in full closure of the center of the contact interface, as highlighted by the TP turning red when the network was fully closed. Finally, the reassembly process finished with global retraction of the lamellipodial structures at the outer cellular periphery, but with a less pronounced general inwards movement of peripheral F-actin (Fig. 1c and Supplementary Movie 1; 32 of the 35 spreading cells).

In this orchestrated series of events, the first two stages were reminiscent of F-actin structures reported during immunological synapse formation in primary mouse embryonic fibroblasts and cytotoxic T lymphocytes^23,25,35,36^, indicating that early events in RBL cell activation are similar to those in non-transformed and cytotoxic T lymphocytes^37,38^. The last two stages were however in stark contrast to F-actin network contractions during immunological synapse formation in lymphocytes, where no symmetry breaking events have been observed and where cellular contraction is more prominent with a clear inward motion of F-actin^26,35^. Notably, as for T cells^23^, RBL cells did not spread in the absence of activating antigens (Supplementary Figure 3).

The processes observed in the last two stages could be a consequence of the central cellular contact region moving out of the axial illumination volume of TIRF. To confirm that the wave-like depletion and reassembly of cortical actin at the basal membrane was indeed governed by F-actin dynamics, we performed 3D *z*-stacks using conventional confocal microscopy. While the membrane was located at the basal plane of the cell at all stages, the central activation zone was indeed depleted of cortical actin (Supplementary Figure 4). Further, we tested whether the F-actin dynamics depended on the stiffness of the activating surface. Qualitatively, no changes in the F-actin dynamics were observed in RBL cells activating on polyacrylamide hydrogels with stiffness of 10 kPa and 100 kPa compared to glass at all activation stages (Supplementary Figure 5).

### Wave-like F-actin structures govern global actin rearrangements

The eTIRF-SIM and 3D-STED images highlighted the rearrangements of the cortical actin cytoskeleton during FCεR-mediated RBL cell activation. To further investigate the processes driving F-actin reorganization, we computed a temporal kymograph along a line at the length axis of the cell (Fig. 1d; same spatial *x*-positions along the line marked in Fig. 1a over time; time as *y*-axis, starting from top). When focusing on the outer and inner edges of the bright F-actin network, one re-identifies the four stages described above. The time-points of symmetry breaking, i.e., network disassembly and reassembly, could clearly be distinguished in the kymograph at time-points of 150 s and 800 s after contact formation (red lines and arrows in Fig. 1d). By tracking the positions of the edges over space and time (Fig. 1d) (or by tracking the cells’ leading edge perimeters over time, Supplementary Figure 2), we determined the velocity of each structure. While cell spreading (leading edge) and network disassembly (inner edges) acted at a mean velocity of *v*_s_ = 58.5 ± 10 nm/s and *v*_dis_ = 81 ± 10 nm/s, respectively, reassembly was around eightfold slower, *v*_re_ = 12 ± 4 nm/s (mean and standard deviation of 35 spreading cells).

### Actin patterns are present during actin rearrangements

At all four stages of activation, very bright actin patches were observed (35 of 35 spreading cells; Fig. 1a–c). During network disassembly and reassembly, the patterns seemingly followed the propagation direction of the F-actin wave-like structure (35 of 35 spreading cells; Fig. 1b, c). At higher magnification, these F-actin structures appeared to have structural similarities with F-actin polymerization patterns, such as vortices and asters (35 of 35 spreading cells; Fig. 1e white and red arrows, respectively), similar to those observed in HeLa cells^17^. To assess the pattern organization of cortical F-actin, we previously developed a robust image analysis pipeline by using a combination of experiments and computational analysis (see Methods). Using this pipeline, we could indeed accurately identify actin patterns in the form of vortices and asters (Supplementary Figure 6). Notably, in the absence of antigens, RBL cells did not generate actin patterns.

These observations led us next to investigate whether actin patterns were involved in the symmetry breaking, and thus the production of the F-actin wave-like structure present during network disassembly and reassembly. For that reason, we carefully monitored the dynamics of the actin patterns in more detail by computing the accumulated number of actin patterns over time (Fig. 1f). The number of patterns peaked at the time-points of symmetry breaking, i.e., the onset of network disassembly coincided with a sharp peak of the number of patterns at 150 s, and was followed by a second and third peak at 800 s and 1000 s just before and during network reassembly (35 of 35 spreading cells). An additional peak was visible at ~1450 s, which could be identified as a transient slowing down of the wave-like propagation in the presented representative images (Supplementary Movie 1). Finally, an overall increase in the number of patterns coincided with the complete network reassembly.

To further understand the interplay of symmetry breaking and actin patterns, and their involvement in wave-like propagation, we localized the individual actin patterns at different time-points, and visualized their spatial distribution as scatter plots (upper panels Fig. 1g, where each spot reveals the position of an individual pattern within the circular cell interface), and as directional histograms (lower panels Fig. 1g and Supplementary Movie 2, where the number of patterns identified within a certain segment of the circular cell are displayed by the lengths of the respective segmental column bars). From these plots, we observed that the patterns accumulated at the opposite edge of the contact interface to where the symmetry broke at *t* = 150 s and *t* = 800 s. In fact, the patterns concentrated at the leading edge and actively followed the propagating wave-like structures during reassembly, for example, at *t* = 1000 s (consistent in 35 of 35 spreading cells). These observations suggested that the actin patterns might be involved in the symmetry breaking events and during F-actin wave-like propagation (Fig. 1g, red arrows). Note that in the following, time-points, *t*, refer to the time between 0 and 60 min from cell contact to network reassembly, and the time-points, *t*', denote events relative to those global time-points, as specified in each case.

### Actin vortices and asters contribute to F-actin propagation

**Fig. 2:**
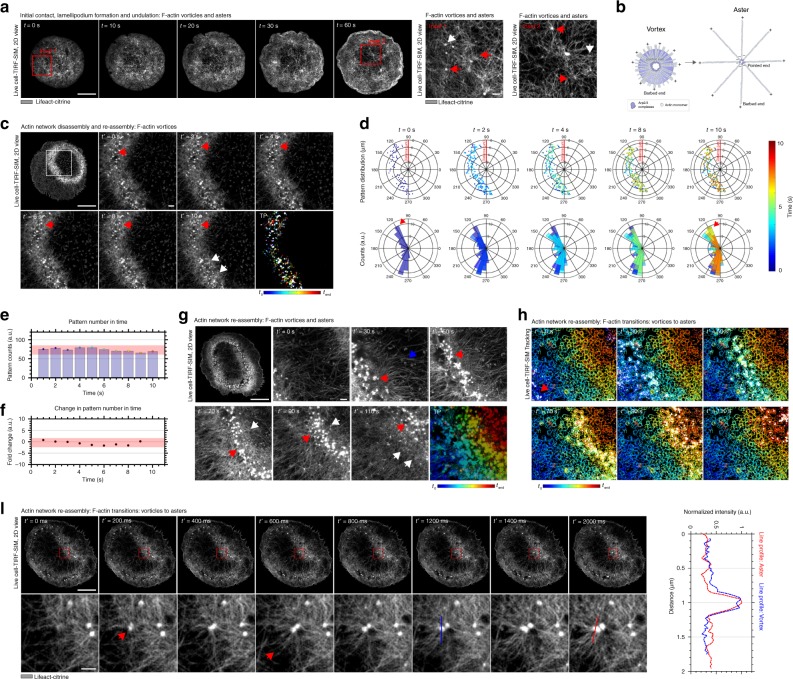
Representative extended total-internal-reflection-fluorescence coupled with structured-illumination microscopy (eTIRF-SIM) experiments of the spatio-temporal dynamics of the actin patterns at the basal membrane during rat basophilic leukemia (RBL) cell activation. **a** Overview (left panels *t* = 0–60 s after contact formation) during initial contact and lamellipodium formation and (insets) zoom-in into red boxes marked at *t* = 0 s (inset 1) and *t* = 60 s (inset 2), highlighting actin asters (red arrows) and vortices (white arrows). Scale bar: 10 μm. **b** Schematic outlining the molecular composition of an actin vortex and aster. **c** Overview during the reassembly process at *t* ≈ 250 s (upper right, scale bar: 10 μm) and zoomed-in snapshots of a high-speed movie (frame rate > 1 Hz) over a short 10 s period taken thereafter (*t*’ = 0–10 s, scale bar: 1 μm) and temporal projections (TPs, lower right), highlighting vortices and asters within (red arrows) and at the peripheries (white arrows). **d** Scatter plots (upper panels) and directional histograms (lower panels) of the pattern positions within the circular cell, **e** total numbers of identified patterns and **f** their change relative to *t*’ = 0 (red shaded area as range of insignificant change, error bars as standard deviation of the mean from 35 images). **g** Overview during the reassembly process at *t* ≈250 s (upper right, scale bar: 10 μm) and zoomed-in snapshots of a medium-speed movie (frame rate 0.1 Hz) over a longer 110 s period taken thereafter (*t*’ = 0–110 s, scale bar: 1 μm) and temporal projections (TPs, lower right) highlighting propagation of patterns with the actin wave, as confirmed by another representation of the zoom-in images in the form of **h** an image of the tracks of each pattern over these 110 s (blue: *t*’ = 0 s to red: *t*’ = 110, the same image in all panels) overlaid by the positions of the patterns (white) at the respective times *t*’. **i** Overviews (upper panels, scale bar: 10 μm) and zoom-ins (lower panels, into red boxes, scale bar: 1 μm) from snapshots taken at different times *t*’ = 0–2000 ms from an ultra-high-speed movie (frame rate 5 Hz) during network reassembly (starting at t ≈ 250 s), indicating transformation of actin vortices into asters (red arrows) at the leading edge of the re-assembling wave-like structure, as further highlighted by (left panel) intensity line profiles through a pattern at *t*’ = 1200 ms (blue, vortex-like) and *t*’ = 2000 ms (red, aster-like) (and by Supplementary Movie 5)

Next, we asked whether the actin patterns played an active role in the global F-actin rearrangements. To this end, we studied the formation and the local organization of the patterns in more detail. Visual inspection of the eTIRF-SIM images allowed us to accurately identify aster- and vortex-like patterns, e.g., by the asterisk-like topology, their dynamics or the vertically outwards radiating F-actin arms in the former case. Note, all results of visual inspections were confirmed by quantitative analysis of the distribution of F-actin orientations^17^. Initial contact formation and spreading coincided with the presence of both actin vortices and asters at all times (35 of 35 spreading cells; Fig. 2a, b and Supplementary Movie 3). In contrast, actin vortices spatially separated from asters during network disassembly and reassembly; while vortices predominately localized to the bulk or center of the wave’s leading edge, asters were located behind the leading edge, as indicated in Fig. 2c and more clearly highlighted in Supplementary Movie 3. High-speed eTIRF-SIM movies taken at a frame rate of <1 s per frame over just 10 s during the reassembly process highlighted that during this short period the actin patterns underwent limited migration yet with some mobility around their median positions (Fig. 2c, TP, Supplementary Movie 3, and Fig. 2d depicting scatter plots (upper panels) and directional histograms (lower panels) of the pattern positions), and that the overall number of patterns was constant (consistent in 32 of 32 spreading cells, Fig. 2e, f). In contrast to the limited dynamics on short time scales, eTIRF-SIM movies recorded over an extended time range of 110 s during network reassembly (taken at higher magnifications and lower temporal resolution, i.e., the usual frame rate of 0.1 Hz) revealed that over this prolonged time period the patterns propagated with the F-actin wave-like structures at its leading edge, as displayed by the directionality of the multiple colors in the temporal projections (Fig. 2g) and by the temporal color-coding of the individual tracking trajectories of the patterns (Fig. 2h and Supplementary Movie 4). Notably, the superposition of these individual tracking trajectories of the patterns appear in the form of cage-like structures surrounding the actin patterns, which was caused by the rotations of the actin vortices during propagation with the F-actin wave-like structure. A closer inspection of the pattern dynamics via ultra-high-speed eTIRF-SIM images taken at a frame rate of 200 ms per frame over a very short period of 2 s during network reassembly further revealed that actin vortices constantly transformed into actin asters at the leading edge, as indicated in Fig. 2i (representative identifications of a vortex and aster shown by intensity line profiles) and more clearly highlighted in Supplementary Movie 5 (consistent in 30 of 30 spreading cells). Quantification of the transition from vortices into asters by visual inspection was technically challenging, and we hence computed the fractions of asters compared to vortices (30 of 30 spreading cells, 96 ± 4% actin patterns were asters) at the leading edge and the center of the wave-like structures (30 of 30 spreading cells, 95 ± 5% actin patterns were vortices).

Conclusively, network disassembly and reassembly were accompanied by the appearance of actin patterns such as vortices and asters, whose dynamics appeared intimately linked to the initial symmetry breaking event and the propagation of network reassembly.

### Arp2/3 but not myosin-II co-localizes with actin patterns

These findings prompted us to examine the molecular players underlying actin pattern reorganization. To this end, we investigated the roles of myosin-II motor proteins and the actin nucleation factor Arp2/3, which have been shown to play a role during self-organized patterning in vitro and in vivo^17,39–43^.

**Fig. 3:**
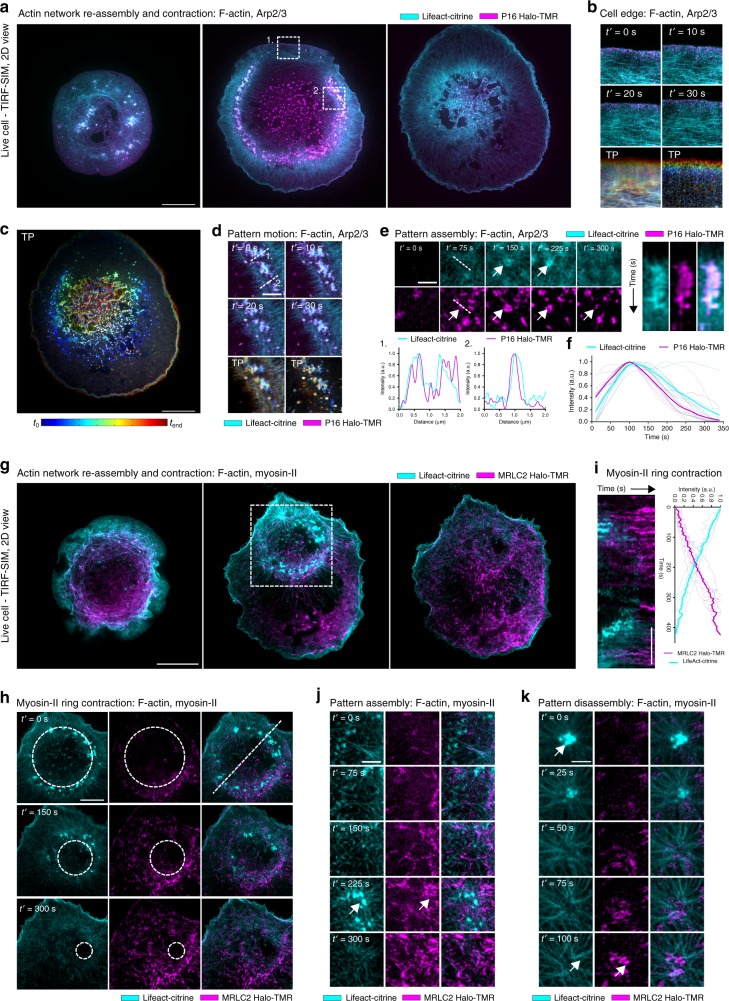
Representative extended total-internal-reflection-fluorescence coupled with structured-illumination microscopy (eTIRF-SIM) experiments of the spatio-temporal dynamics of the F-actin network and patterns (Lifeact-citrine) together with Arp2/3 complexes (p16-Halo with TMR dye-ligand) and myosin-II (MRLC2-halo with TMR dye-ligand) at the basal membrane during rat basophilic leukemia (RBL) cell activation. **a** Snapshots showing the distribution of F-actin (cyan) and Arp2/3 (magenta) during contact formation, network disassembly and network reassembly. Scale bar: 10 μm. **b** Zoom-in and temporal projection (TP) of inset region 1. in **a** of Arp2/3 localization at the leading edge of the lamellipodium during contact formation. Total time is 500 s. Scale bar: 2 μm. **c** Temporal projection of Arp2/3 localization during network reassembly. **d** (Left panels) Zoom-in and TP of inset region 2 in **a** of actin pattern progression showing the high degree of co-localization of actin and Arp2/3. Scale bar: 2 μm. **d** (Right panels) Representative line profiles further highlighting the co-localization. **e** (Left panels) Zoom-in showing a representative time-lapse of pattern assembly. An increased in Arp2/3 intensity precedes actin pattern formation. Scale bar: 1 μm. **e** (Right panels) Kymograph corresponding to **e** highlighting the spatio-temporal co-localization of Arp2/3 and F-actin during pattern assembly. Total time is 300 s. **f** Mean line-profile showing the relative intensity of F-actin and Arp2/3 over time during pattern assembly, indicating that Arp2/3 localization precedes actin nucleation. **g** Snapshots showing the distribution of F-actin (cyan) and myosin-II (magenta) during contact formation, network disassembly and network reassembly. Scale bar: 10 μm. **h** Zoom-in highlighting the formation and progression of the myosin-II ring structure surrounding actin patterns during network reassembly of boxed region in **g**. As the actin pattern progressed, myosin-II localization follows behind the leading edge, eventually filling the previously actin-depleted zone. Scale bar: 5 μm. **i** (Left panel) Kymograph showing the formation and progression of the myosin-II ring structure surrounding actin patterns during network reassembly. Total time is 400 s. Scale bar: 5 μm. **i** (Right panel) Line plot showing the mean actin and myosin-II intensity over time during network reassembly. **j** Zoom-in showing the localization of myosin-II in the periphery during pattern assembly. Scale bar: 1 μm. **k** Zoom-in showing the localization of myosin-II during pattern disassembly. Scale bar: 1 μm

To assess the role of Arp2/3 in pattern organization and F-actin wave-like structure propagation, we fluorescently labeled F-actin (Lifeact-citrine) and Arp2/3 (via p16 or ARPC5, one of the seven subunits of the Arp2/3 complex^44^, using a Halo-Tag and membrane-permeable tetramethylrhodamine (TMR) dye-ligand). As expected from previous observations in live HeLa cells^17^, the Arp2/3 complexes spatially co-localized with the actin patterns, as representatively depicted in the eTIRF-SIM images taken during the initial contact formation, network disassembly and reassembly (Fig. 3a and Supplementary Movie 6). Further, our images highlight the typical predominant localization of the Arp2/3 complex to the leading edge of the lamellipodium (Fig. 3b and Supplementary Movie 7). During the wave-like progression of patterns upon network reassembly, Arp2/3 displayed clear foci that correlated with F-actin structures, as highlighted by the temporal projection in Fig. 3c and dual color line profiles in Fig. 3d (Pearson’s correlation coefficient of 0.56 ± 0.11, mean and standard deviation of 10 cells) (see also Supplementary Movie 8). Consistent with ref. ^17^, this suggested that the Arp2/3 complex was involved in the organization of the actin patterns. By analyzing individual pattern formation, it was possible to observe the temporal series of events leading to formation (Fig. 3e). By taking kymographs and corresponding line profiles, it was evident that a local increase in Arp2/3 intensity is quickly followed by an increase in F-actin intensity, further supporting the hypothesis that Arp2/3 nucleation is involved in pattern formation (Fig. 3f).

To assess the role of myosin-II in pattern organization and F-actin wave-like structure propagation, we fluorescently labeled F-actin (Lifeact-citrine) and myosin-II motor proteins (MRLC2 Halo-Tag and membrane-permeable TMR dye-ligand). Myosin-II was minimally present at the cell interface during spreading and F-actin network disassembly but became more prominent during reassembly (22 of 22 spreading cells; Fig. 3g). Specifically, at the beginning of this process (*t*’ = 0–60 s) the myosin-II motors randomly localized to F-actin in the central regions of the cell rather than to peripheral F-actins, and they did not overlap with the actin patterns (Pearson’s correlation coefficient of −0.02 ± 0.07, mean and standard deviation of 7 cells), consistent with previous observations in live HeLa cells^17^. Most strikingly, following the symmetry breaking event within the actin cortex, myosin-II motors localized in a ring surrounding the actin patterns (Fig. 3h and Supplementary Movie 9). As the actin patterns progressed, closing the central zone, the myosin-II ring advanced to follow the actin patterns. At the completion of network reassembly, myosin-II was localized across the cell interface. In general, myosin-II localization increased linearly as the cell contact matured during activation, which negatively correlated with the intensity of F-actin, as quantified in Fig. 3i. By close inspection of high-speed eTIRF-SIM time-lapse imaging, we next sought to investigate the role of myosin-II in the assembly and disassembly of the actin patterns. Figure 3j shows a representative time-lapse of pattern assembly, showing again that myosin-II displays poor localization with the patterns themselves, but forms structures which surround the patterns (see also Supplementary Movie 10). This is likely due to binding to the high density of newly polymerized F-actin formed during pattern assembly. Intriguingly, when myosin-II localization reaches its maximum, the patterns disassemble rapidly, suggesting an important role for myosin-II in pattern maturation and disassembly. This is further highlighted in Fig. 3k, where pattern disassembly correlates with an increase in myosin-II localization in the periphery of the pattern (see also Supplementary Movie 11).

The different roles of myosin-II and the Arp2/3 complex in the formation and maintenance of self-organized actin patterns during FCεR-mediated RBL cell activation are further highlighted by pharmacological treatments. Cytoskeletal rearrangements arrested and no contact interface was formed when myosin motor activity was blocked with 100 µM of the Rho-kinase inhibitor Y27632 prior to contact formation (24 of 24 spreading cells; Supplementary Figure 7A). Similarly, specific inhibition of the Arp2/3 complex with 100 µM CK666 prior to spreading ceased contact formation, and discontinued subsequent F-actin rearrangements (25 of 25 spreading cells; Supplementary Figure 7B). CK666-induced inhibition of the Arp2/3 nucleation activity at *t* = 180 s after initial contact formation (i.e., prior to the first symmetry breaking event) completely arrested the expected subsequent F-actin cytoskeleton rearrangements, specifically the lamellipodial undulations and the central F-actin disassembly and reassembly and general F-actin network dynamics (22 of 22 spreading cells; Supplementary Figure 7C,D kymographs). Instead, the treatment resulted in an isotropically distributed cortical network at the contact interface that was dominated by asters (but no vortices, Supplementary Figure 7C, red arrows). The latter distinct formation of actin asters upon perturbation of the Arp2/3 complex was consistent with observations of actin patterns in HeLa cells after Arp2/3 inhibition^17^. Note that the corresponding dimethyl sulfoxide controls did not yield any changes in the F-actin rearrangements compared to control conditions. Together, these data suggested that the Arp2/3 complex but not myosin-II plays a role in the nucleation of actin vortices and asters, whereas myosin-II may play an important role in the progression of the patterns and their disassembly.

### Cytoskeletal actin patterns and FCεR organization

We next explored the potential relationship between F-actin wave-like propagation and centripetal reorganization of FCεR clusters at the RBL cell interface during activation by fluorescently labeling F-actin (Lifeact-citrine) and the FCεR clusters (SNAP-Tag and membrane-permeable TMR dye-ligand). To follow both fluorescence channels over longer periods of time, we turned to confocal Airyscan microscopy (Supplementary Figure 8A), allowing us to track both the F-actin wave-like structures and the positions of FCεR clusters. During network reassembly, the FCεR clusters moved freely within the central F-actin-free zone, while the area they were allowed to explore became more restricted as the F-actin structures moved inwards, closing the actin-free zone. Consequently, the FCεR clusters displayed an overall trend to precede the centrally propagating F-actin wave-like structure during F-actin reassembly. This is further confirmed by the fact that the velocity with which the F-actin wave-like structure moved inwards (*v*_re_ = 12 ± 4 nm/s, compare Fig. 1d) was comparable (*p* = 0.98) with that of the FCεR clusters in the decrease of the area in which they were allowed to move freely (*v*_FCεR_ = 10 ± 4 nm/s), i.e., the F-actin wave-like structure seemed to restrict and direct the trajectories of the FCεR clusters (21 of 21 spreading cells).

### Cortical actin network dynamics and granule exocytosis

Finally, we sought to understand the physiological role of F-actin disassembly and reassembly. The activation of RBL cells through the interaction of IgE with FCεR leads to an immune response in form of the exocytosis of histamine-rich granules. It has previously been shown that vesicle secretion in FCεR-mediated RBL cell activation coordinates with levels of cortical actin^45^. Given these observations, it becomes apparent that vesicle secretion might also coordinate with the disassembly and reassembly processes reported here. Hence, we fluorescently labeled F-actin (Lifeact-citrine) and intracellular vesicles (using Annexin V-Alexa 647^46^) and imaged their dynamics during RBL cell activation using two-color eTIRF-SIM. Annexin V has been shown to specifically bind to cell surface secretory granules in proportion to the degree of degranulation due to its interaction with phosphatidylserine exposed on the plasma membrane during secretion^47^.

**Fig. 4:**
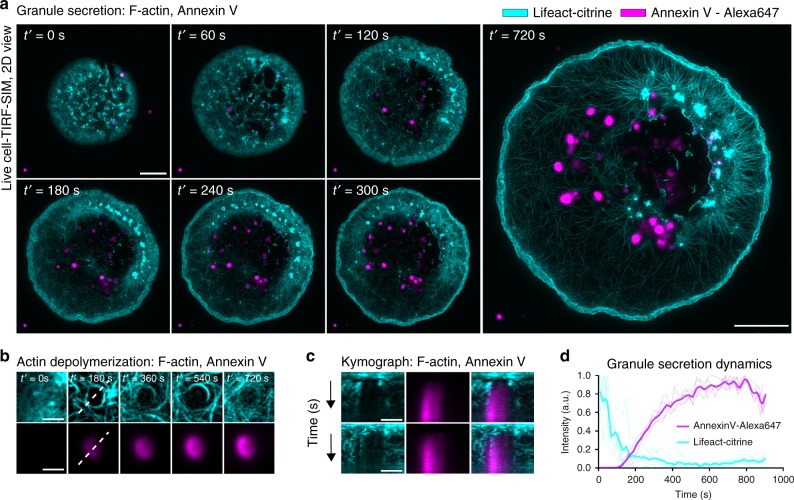
Representative extended total-internal-reflection-fluorescence coupled with structured-illumination microscopy (eTIRF-SIM) experiments of the spatio-temporal dynamics of the F-actin network and patterns (Lifeact-citrine) together with granule secretion (Annexin-V-647) at the basal membrane during rat basophilic leukemia (RBL) cell activation. **a** Snapshots during contact formation and subsequent F-actin network disassembly and reassembly (cyan) showing the increased formation of zones rich in Annexin V staining (magenta), indicative of granule secretion. Scale bar: 5 μm. **b** Zoom-in of F-actin dynamics during granule secretion. Disassembly of the actin cortex coincides with increased binding of Annexin V, indicating that F-actin dynamics is critical for the granule release. Scale bar: 1 μm. **c** Two representative kymographs highlighting the rapid de-polymerization of F-actin followed by the gradual increase in Annexin V intensity. Total time is 720 s. **d** Mean line-profile showing the temporal progression of F-actin network de-polymerization and Annexin V staining

During RBL cell spreading, the number of Annexin V-rich area increased (Fig. 4a and Supplementary Movie 12). The areas rich in Annexin V binding localize within the ring of actin patterns that formed at the contact interface, suggesting that F-actin rearrangements were crucial in allowing exocytosis of granules during RBL cell activation. Close examination of an individual Annexin V-rich area revealed a striking relationship to the dynamics of the actin cytoskeleton. Initially, the actin cortex formed a dense mesh, but abruptly formed a small (~1 µm) circular opening, which remained stable for the duration of the activation process (Fig. 4b and Supplementary Movie 13). After the opening of the F-actin meshwork, we observed a gradual increase in the intensity of Annexin V staining, suggesting that the opening in the actin cortex enabled granule secretion. This could be further visualized using kymograph and line-profile analysis, which showed a clear drop in the F-actin intensity (opening of the cortex) followed by a gradual increase in the Annexin V intensity over time (Fig. 4c, d).

## Discussion

Using advanced optical microscopy in live RBL cells, we demonstrated how cytoskeletal actin patterns characterize RBL cell activation. Super-resolved microscopy data of the cortical actin interface revealed the presence of actin vortices and asters, which appeared to orchestrate subsequent stages of RBL cell activation. Importantly, F-actin reorganization coincided with apparent granule secretion as well as the organization of FCεR clusters towards the interface center. In support of this observation, recent work has shown that the organization of the actin cytoskeleton plays a critical role in enabling the secretary function of natural killer cells and cytotoxic T lymphocytes^22^. These observations were generally in line with previous investigations in RBL cells, such as that levels of F-actin correlated with calcium responses and cytoplasmic granule exocytosis^45,48–50^.

While self-organizing actin patterns have long been known from in silico considerations^21^ and in vitro experiments^19,20^, they have only recently been directly imaged in living cells^17^. The actin patterns detected here in live RBL cells show high similarities with the previously described patterns. Closer inspection of our current RBL cell data highlighted that the propagation of the F-actin wave-like structures followed the fast and continuous transformations between the different actin patterns, specifically of vortices into asters. Transformations from vortices into asters were also observed in the previous HeLa cell recordings, specifically during de novo cortex formation^17^, but also resulted in the formation of another, larger actin pattern, namely stars, which was not observed here.

Pharmacological perturbation during the activation of RBL cells indicated that the F-actin reorganization and pattern formation was reliant on the Arp2/3 complex and further assisted by myosin-II. Additional mechanistic studies will be required to fully elucidate the mechanistic role of the Arp2/3 complex and motor proteins in the formation of F-actin wave-like structures and actin pattern formation. Quantifying the dynamics of the actin patterns allowed us to identify an orchestrated cascade of events that suggests the relevance and impact of these patterns to the activation process of RBL cells.

Together with knowledge from theoretical considerations and studies in vivo and in situ (see Introduction), we speculate that actin patterns result from changes in the intrinsic mechanical stress of F-actin networks. We assume that tuning the nucleation activity of the Arp2/3 complex controls the abundance of the actin patterns, because pharmacological inhibition of the Arp2/3-mediated F-actin assembly suppressed pattern formation in RBL cells, and enforced transformations in between actin patterns from actin stars to asters in HeLa cells, assuming that they rely on the same processes^17^. Nevertheless, it remains unclear how the cells achieved the symmetry breaking events during activation, although it is likely that there is a relationship with the occurrence of the total number of actin patterns, which coincided with the time-points of the symmetry breaking events. Notably, the symmetry breaking event could be either a cause or a consequence of actin pattern formation. To prove every element of the outlined processes, the intrinsic mechanical stress throughout the cortical F-actin network needs to be monitored simultaneously to the F-actin dynamics, as well as the localization dynamics of the Arp2/3 complex and associated players at extended spatial-temporal resolution. Measuring this mechanical stress and the required energy consumption during the evolution of the F-actin network would provide further details of the identified process, and ultimately demonstrate how cytoskeletal actin patterns form and disassemble under such circumstances.

In the future, advanced microscopy such as super-resolution techniques may reveal the wider importance of the self-organization of the actin cytoskeleton^33,51,52^. Considering the importance and implications of actin self-organization in the current case of the activation of immune responses, future research should focus on the nanoscale organization of actin networks from the bottom up. To this end, all three inherently interconnected components of the cortical actin cytoskeleton, namely actin turnover kinetics, network dynamics and mechanics, should be considered, knowing that most immune cells share very similar actin signaling pathways and identical actin nucleators^53,54^. Yet, these pathways and nucleators may still give rise to different F-actin organization principles such as self-assembly and self-organization^53,54^.

In summary, we have demonstrated the emergence of cytoskeletal actin patterns in live immune RBL cells. This was made possible due to the use of advanced optical microscopy techniques, whose use will hopefully allow further observations of actin self-organization in living cells and the investigation of their roles in cellular signaling.

## Methods

### Cell culture

RBL-2H3 clone cells (CRL-2256, ATCC, USA) were cultured at 37 °C in 5% CO_2_ in Minimum Essential Media (MEM) (Sigma Aldrich) containing 15% fetal bovine serum (FBS), 10 mM HEPES (Lonza, UK), 1% penicillin–streptomycin and 1% l-glutamine. Cells were split every 2 days at a volume ratio of 1:5. At 24 h prior to experiments, adherent cells were treated with 0.05% Trypsin-EDTA (Lonza), facilitating their detachment from the cell culture flask. Cells were then transferred to a rotating chamber at 37 °C in 5% CO_2_ to maintain their suspension state prior to the experiments.

### Plasmids

We obtained vectors encoding C-terminal tagged Lifeact-citrine^17^, p16-halo^17^, MRLC2-halo^17^ and FCεRI-snap^27^ via PCR amplification of the respective genes. This produced double-stranded DNA fragments encoding Lifeact, p16, MRLC2 and FCεRI sequences followed by a Gly-Ser linker and flanked by 5′ *Mlu*I and 3′ *Bam*HI restriction nuclease sites. Following digestion with *Mlu*I and *Bam*HI, we ligated these fragments into pHR-SIN lentiviral expression vectors containing the Citrine, halo or snap gene downstream of the *Bam*HI site in the correct reading frame. Sequence integrity was confirmed by reversible terminator base sequencing.

### Generation of stable cell lines

We used a lentiviral transduction strategy to generate RBL cell lines stably expressing Lifeact-citrine, p16-halo, MRLC2-halo and FCεRI-snap. We plated HEK-293T cells in 6-well plates at 3 × 10^5^ cells per mL, 2 mL per well in Dulbecco's modified Eagle's medium (DMEM)+10% FBS. Cells were incubated at 37 °C and 5% CO_2_ for 24 h before transfection. Transfection was performed with 0.5 μg per well of each of the lentiviral packaging vectors p8.91 and pMD.G and the relevant pHR-SIN lentiviral expression vector using GeneJuice (Merck Millipore), as per the manufacturer’s instructions. At 48 h post transfection, we harvested the cell supernatant and filtered using a 45 μm Millex-GP syringe filter unit to remove detached HEK-293T cells. Then, 3 mL of this virus-containing medium was added to 1.5 × 10^6^ RBL cells in 3 mL supplemented RPMI-1640 medium. After 48 h, we moved cells into 10 mL supplemented MEM and passaged as normal.

### Microscope coverslide preparation

To prepare IgE-coated coverslips, we coated coverslips with TNP-BSA at a concentration of 500 µg per mL and incubated at 4 °C overnight. Coverslips were then washed 3 times using phosphate-buffered saline (PBS), followed by the addition of a 10% solution of BSA for a further 1 h at room temperature. Following this, coverslips were washed 3 times in PBS and then coated with a 5 μg per mL IgE (Clone IgE-3, BD Biosciences) for 1 h at room temperature. Following incubation, coverslips were washed a further 3 times in PBS before being used at the microscope.

### Polyacrylamide gel preparation

Polyacrylamide gels were prepared as previously described^55^. Briefly, 10 kPa and 100 kPa poly(acrylic acid) (PAA) gels were prepared by combining acrylamide monomers (Sigma Aldrich) at 10% and varying the concentration of bis-acrylamide cross-linkers (Sigma Aldrich) from 0.1% to 0.4%, respectively. Polymerization was initiated by the addition of TEMED (Sigma Aldrich) followed by 10% Ammonium persulfate (Sigma Aldrich) at a volume ratio of 1:250 and 1:100, respectively. The gel solution was pipetted between two glass coverslips, one of which having been treated by APTMS 0.5% (Sigma Aldrich) followed by 0.5% glutaraldehyde (Sigma Aldrich) to firmly attach the gel to the coverslip.

PAA functionalization was achieved using the ultraviolet (UV) activated cross-linker Sulfo-SANPAH (Thermo Fischer). Each gel was coated with a 20 mg per mL solution of Sulfo-SANPAH and exposed to 365 nm UV light for 10 min. The gel was then washed to remove any excess cross-linker and then coated with a 500 µg per mL solution of TNP-BSA and incubated at 4 °C for 12 h. Gels were then washed and coated with a 10 µg per mL IgE anti-TNP solution for 1 h at room temperature, followed by a further washing step.

### High-NA eTIRF-SIM microscopy

The high-NA (high-numerical-aperture) eTIRF-SIM was performed using a custom-built system described in detail in ref. ^33^. Structured illumination was achieved using a grating pattern generated by a ferroelectric spatial light modulator (SLM, Forth Dimension Displays, SXGA-3DM). Sample illumination was carried out using a high-NA objective (Olympus Plan-Apochromat 100X Oil-HI 1.57NA or Olympus Plan-Apochromat 100X 1.49NA) in TIRF mode, penetrating light ~100 nm light into the sample. The emitted fluorescence was collected by the same objective and imaged onto an sCMOS camera (Hamamatsu, Orca Flash 4.0 v2 sCMOS), where the structured fluorescence raw data were recorded. All live-cell imaging was performed at 37 °C and 5% CO_2_. A total of 9 raw images was acquired for a single excitation wavelength before switching to the next, and then repeating this acquisition procedure for each time point. Finally, the raw images were processed and reconstructed into SIM images by a previously described algorithm^56^.

We used the ImageJ plugin ‘JACoP (Just Another Colocalization Plugin)’ (ImageJ, http://imagej.nih.gov/) for fluorescence overlay analysis. Notably, we calculated the Squared-overlap-coefficient *r*^2^ and Pearson’s correlation coefficients instead of the conventional-overlap coefficient to minimize the effects of background and zero intensity values in one of the two channels as outlined in ref. ^57^. eTIRF-SIM data are presented in Figs. 1–4 and Supplementary Figures 3, 7, 8 with the exact frame rates given in the captions and text. For each experimental condition, eTRIF-SIM data were acquired in 12–45 individual cells over the course of at least 3 independent experiments.

### Drug treatments

Pharmacological actomyosin-specific reagents CK666 and Y27632 (Merck Bio-sciences, UK) were added to the culture medium at the given concentrations and the cells were left to incubate between 30 s and 30 min, as indicated in the corresponding experiment description. Notably, inhibitors were also present at the same concentration in the imaging medium. Drug treatment experiments are presented in Supplementary Figure 7. In all cases, we controlled that the cells did not show changes following the same protocols without the added drugs.

### Granule exocytosis quantification

Granule exocytosis was imaged using a previously described protocol^46^. Briefly, imaging medium was prepared using Tyrodes buffer containing 1% vol/vol Annexin V-Alexa 647 stain (A23204, Thermo). RBL cells were allowed to activate on coming into contact with IgE functionalized coverslips in the presence of the described imaging medium.

### Orientation analysis

The simulated fiber geometries of vortices and asters were computed in custom-written MATLAB routines (MATLAB Inc, UK), as further outlined in ref. ^17^. Using the ‘OrientationJ' Java plugin for ImageJ (http://imagej.nih.gov/), the distribution of fiber orientations within a region of interested was evaluated, as outlined in www.epfl.ch/demo/orientation/. Data presentation was performed in custom-written MATLAB routines (MATLAB Inc.). Orientation analysis is presented in Supplementary Figure 6.

### Temporal projections

To calculate the TP from eTIRF-SIM time-lapses as shown in Figs. 1a–c, 2b, f, 3b, c, d, a multi-stage image-processing protocol was performed. The ImageJ plugin ‘Temporal color-code' (ImageJ, http://imagej.nih.gov/) was used to superimpose all images of a time-lapse onto one plane coding each frame with cold to warm colors representing early to late time-points. Custom-written ImageJ LUTs (look up tables) were employed to represent early time frames in blue and late ones in red.

### STED microscopy

STED experiments were performed on a Leica TCS SP8 3X microscope (Leica, Mannheim, Germany), as described in detail in ref. ^17^. STED imaging of Lifeact-citrine was performed using 488 nm excitation light in combination with 592 nm STED depletion light. Laser powers were tuned to obtain a strong enough fluorescence signal as well as sufficient improvement in spatial resolution, and images were acquired at 1–5 s intervals to minimize loss of fluorescence due to photo-bleaching as well as cell phototoxic effects (of which we did not observe any in the recordings). All live-cell experiments were performed at 37 °C and 5% CO_2_.

STED imaging was post-processed using the Huygens STED-Deconvolution-Wizard (Huygens Software, The Netherlands), as described in ref. ^17^, where only a moderate degree of deconvolution was applied to the recorded STED images to avoid deconvolution artifacts. STED experiments are presented in Supplementary Figure 1. STED microscopy was performed in at least 25 individual cells for each experimental condition over the course of at least 3 independent experiments.

### Airyscan microscopy

Airyscan imaging was performed with a confocal laser scanning microscope ZEISS LSM 880 with an alpha Plan-Apochromat 63 × /1.46 oil objective. The microscope was additionally equipped with Airyscan detection module (Zeiss, Oberkochen, Germany)^58^. All live-cell experiments were done at 37 °C and 5% CO_2_. A diode laser at 561 nm and Argon laser light at 488 nm were used as fluorescence excitation sources. Excitation powers were set at 1–3% for both lasers (1–5 µW), and were adjusted within this range for each image individually in order to achieve a strong enough fluorescence signal for both channels. Fluorescence emission was collected at around 595 nm and 515 nm for the red and green channels, respectively, with the filters BP570–620+LP645 (red) and BP420–480+BP495–550 (green). The image time series were acquired with 20 s time interval between the measurements to minimize photo-bleaching and photo-toxicity. The emission signals for both channels were collected subsequently on the 32 channel GaAsP-PMT Airy detector^58^. ZEN airyscan software (Zeiss) was used to process the acquired data sets. This software processes each of the 32 Airy detector channels separately by performing filtering, deconvolution and pixel reassignment in order to obtain images with enhanced resolution and improved signal to noise ratio. The value of the Wiener filter in ZEN software was set between 6 and 7 to avoid deconvolution artifacts, but to ensure up to 1.5× resolution improvement. Airyscan time series measurements were performed in at least 12 individual cells for each experimental condition over the course of 5 independent experiments. Airyscan microscopy data are presented in Supplementary Figure 8. Detailed information for optimal Airyscan microscopy is discussed in ref. ^59^.

### Pattern tracking

Using custom-written MATLAB routines, eTIRF-SIM images were segmented by manually localizing the actin vortices and asters in the raw microscope images. For each frame of the time-lapse, the *x*–*y* position of each pattern was recorded and subsequently tracked, allowing the number and spatial position of the patterns to be quantified over time. Spatial positions were then plotted onto scatter plots and scatter-histograms in time, whereas color-coding from blue to red reported on early to late time-points. Scatter-histogram were computed by binning 12 degrees from 0 to 360 degrees. Pattern tracking was employed in Fig. 2g and Supplementary Figure 8.

### Statistics

Statistical analysis of the data was performed with standard *t*-test, whereas *p* values were significantly different for *p* < 0.01. All experiments were performed in at least 12 individual cells for each experimental condition over the course of at least 3 independent experiments.

### Reporting summary

Further information on experimental design is available in the Nature Research Reporting Summary linked to this article.

## Supplementary information

Description of Additional Supplementary Files

Reporting Summary

Supplementary Movie 11

Supplementary Movie 12

Supplementary Movie 13

Supplementary Movie 1

Supplementary Movie 2

Supplementary Movie 3

Supplementary Movie 4

Supplementary Movie 5

Supplementary Movie 6

Supplementary Movie 7

Supplementary Movie 8

Supplementary Movie 9

Supplementary Movie 10

Supplementary Information

## Data Availability

Any data generated or analyzed during this study that are not included in the published paper or its supplementary information files are available from the authors upon request.
