# Supplementary material for: Cytoskeletal actin patterns shape mast cell activation
Source: Commun Biol. 2019 Mar 7;2:93. doi: 10.1038/s42003-019-0322-9 (PMC6405992; doi:10.1038/s42003-019-0322-9)
Supplement: Supplementary file 1 — Description of Additional Supplementary Files [file 42003_2019_322_MOESM1_ESM.pdf]

Supplementary Movies

Movie S1: Extended TIRF-SIM movie of the basal plane of a representative RBL cell expressing LifeAct-citrine on activating glass coated with TNP-BSA IgE. The movie shows in the xy-view the cell with the glass at the bottom. Due to the high dynamic range of the movie, faint actin structures are shown in the left panel, while bright actin pattern are shown in the right panel. RBL cells formed a symmetric interface upon initial contact with the activating surface, centripetally growing into an undulating lamellipodium. Symmetry of the cortical actin network spontaneously broke and a non-symmetric actin-depolymerization wave traveled from the lower left of the contact interface to the upper right. Once completely disassembled, the actin-depolymerization wave turned into a polymerizing one, thus changing traveling direction at 180 degrees, and propagating back to its initial position. Scale-bar: 10 µm. The time between frames is 10 s and the total duration ~40min.

Movie S2: Tracking quantifications of actin patterns in representative RBL cell expressing LifeAct-citrine on activating glass coated with TNP-BSA IgE. The movie shows the development of the distribution of actin vortices and asters (left scatter-plot and histogram-plot) during activation.

Movie S3: Extended TIRF-SIM movie of the basal plane of a representative RBL cell expressing LifeAct-citrine on activating glass coated with TNP-BSA IgE. The movie shows in the xy-view the cell with the glass at the bottom. Little directed motion was visible at those high-speed temporal resolutions. Scale-bar: 5 and 1 µm. The time between frames is 1 s and the total duration ~40min.

Movie S4: Tracking of actin patterns presented in Movie S3. Color-coding represents early to late time-points from cold to warm color. Scale-bar: 1 µm. The time between frames is 1 s.

Movie S5: Extended TIRF-SIM movie of the basal plane of a representative RBL cell expressing LifeAct-citrine on activating glass coated with TNP-BSA IgE. The movie shows in the xy-view the cell with the glass at the bottom. Continuous transformations from actin vortices into asters are visible. Scale-bar: 5 and 1 µm. The time between frames is 200 ms.

Movie S6: Extended TIRF-SIM movie of the basal plane of a representative RBL cell expressing LifeAct-citrine (cyan) and p16 with Halo-Tag and TMR dye-ligand (magenta) on activating glass coated with TNP-BSA IgE. The movie highlights the high degree of co-localization between actin patterns and Arp2/3 during network reassembly. Scale-bar: 10µm. The time between frames is 10 s and the total duration ~8min.

Movie S7: Extended TIRF-SIM movie of the basal plane of a representative RBL cell expressing LifeAct-citrine (cyan) and p16 with Halo-Tag and TMR dye-ligand (magenta) on activating glass coated with TNP-BSA IgE. The movie highlights the localization of Arp2/3 at the leading edge of the activating RBL cell during contact formation. Scale-bar: 2µm. The time between frames is 2 s and the total duration ~40s.

Movie S8: Extended TIRF-SIM movie of the basal plane of a representative RBL cell expressing LifeAct-citrine (cyan) and p16 with Halo-Tag and TMR dye-ligand (magenta) on activating glass coated with TNP-BSA IgE. Zoom-in highlights the high degree of co-localization between actin patterns and Arp2/3 during pattern progression. Scale bar: 2µm. The time between frames is 2 s and the total duration ~40s.

Movie S9: Extended TIRF-SIM movie of the basal plane of a representative RBL cell expressing LifeAct-citrine (cyan) and MRLC2 with Halo-Tag and TMR dye-ligand (magenta) on activating glass coated with TNP-BSA IgE. The movie highlights the formation of a myosin-II ring surrounding the actin patterns during network reassembly. On full network reassembly, myosin-II localizes across the contact area. Scale bar: 5μm. The time between frames is 5 s and the total duration ~7min.

Movie S10: Extended TIRF-SIM movie of the basal plane of a representative RBL cell expressing LifeAct-citrine (cyan) and MRLC2 with Halo-Tag and TMR dye-ligand (magenta) on activating glass coated with TNP-BSA IgE. Zoom-in highlights the localization of myosin-II in the periphery of the actin patterns during pattern assembly. Scale bar: 1μm. The time between frames is 5 s and the total duration ~7min.

Movie S11: Extended TIRF-SIM movie of the basal plane of a representative RBL cell expressing LifeAct-citrine (cyan) and MRLC2 with Halo-Tag and TMR dye-ligand (magenta) on activating glass coated with TNP-BSA IgE. Zoom-in highlights the possible role of myosin-II in the disassembly of actin patterns, showing increased localization in the pattern periphery during disassembly. Scale bar: 1μm. The time between frames is 5 s and the total duration ~7min.

Movie S12: Extended TIRF-SIM movie of the basal plane of a representative RBL cell expressing LifeAct-citrine (cyan) and labelled with AnnexinV-647 on activating glass coated with TNP-BSA IgE. The movie indicates the appearance of distinct regions showing a gradual increase in AnnexinV binding during network disassembly. Scale bar: 5μm. The time between frames is 20 s and the total duration ~8min.

Movie S13: Extended TIRF-SIM movie of the basal plane of a representative RBL cell expressing LifeAct-citrine (cyan) and labelled with AnnexinV-647 on activating glass coated with TNP-BSA IgE. Zoom-in of Movie S12 highlighting the co-localization of the depolymerization of the actin cortex and the appearance of AnnexinV binding regions. Scale bar: 1μm. The time between frames is 20 s and the total duration ~8min.
