# Supplementary material for: Cytoskeletal actin patterns shape mast cell activation
Source: Commun Biol. 2019 Mar 7;2:93. doi: 10.1038/s42003-019-0322-9 (PMC6405992; doi:10.1038/s42003-019-0322-9)
Supplement: Supplementary file 16 — Supplementary Information [file 42003_2019_322_MOESM16_ESM.pdf]

Supplementary Figures

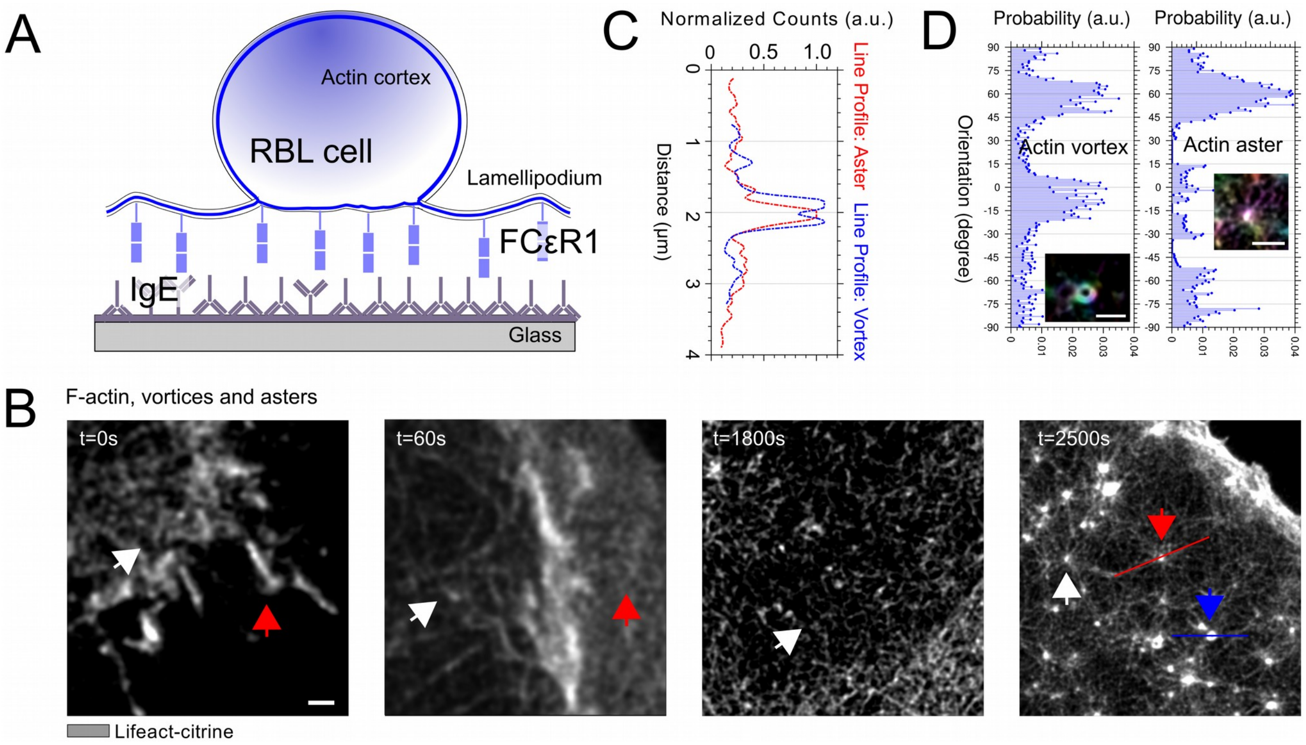

Supplementary Figure 1: Characterization of self-organizing actin patterns in RBL cell activation.

(A) Schematic of RBL cells expressing FCεR that interact with activating microscope cover-glass frictionalized with TNP-BSA crosslinked IgE. (B) Representative images acquired with 3D Stimulated Emission Depletion (STED) microscopy. STED was applied to acquire images of the basal cytoskeletal reorganizations in RBL cells when making initial contact to surfaces coated with activating antibodies. F-actin of RBL cells was labelled with Lifeact-citrine. RBL cells formed a contact with the surface (t=0s) with a dense actin network at its contact centre (white arrow) and longer F-actin in the periphery; lower panel, red arrow), followed by the formation and undulation (t=60s) of a lamellipodium with very dense F-actin network (red arrow) surrounding a coarser centric network. The centric network displayed a particuarly coarse region in its middle (white arrow) suggesting some degree of cytoksleletal disassembly and re-assembly (t=1800s). Finally, the lamellipodium retracted (t=2500s), showing actin patterns such as vortices (red arrow) and asters (white arrow). Scale-bar: 10µm and 1µm.

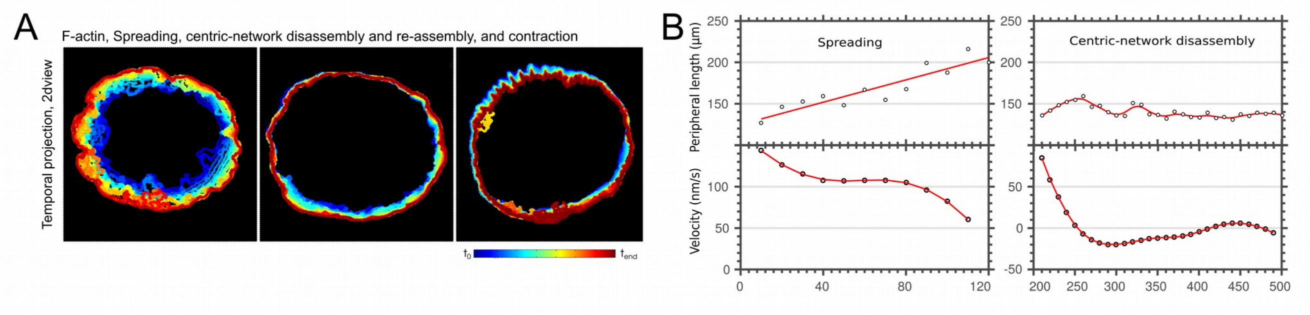

Supplementary Figure 2: Tracking the periphery of the leading edge of the cell contact as presented in Fig. 2. (A) Early times are represented with cold colours and later times with warm colors. (B) Corresponding graph of the peripheral length in radial direction of the contact interface (top panel) and velocity (bottom panel) during spreading and centric network disassembly.

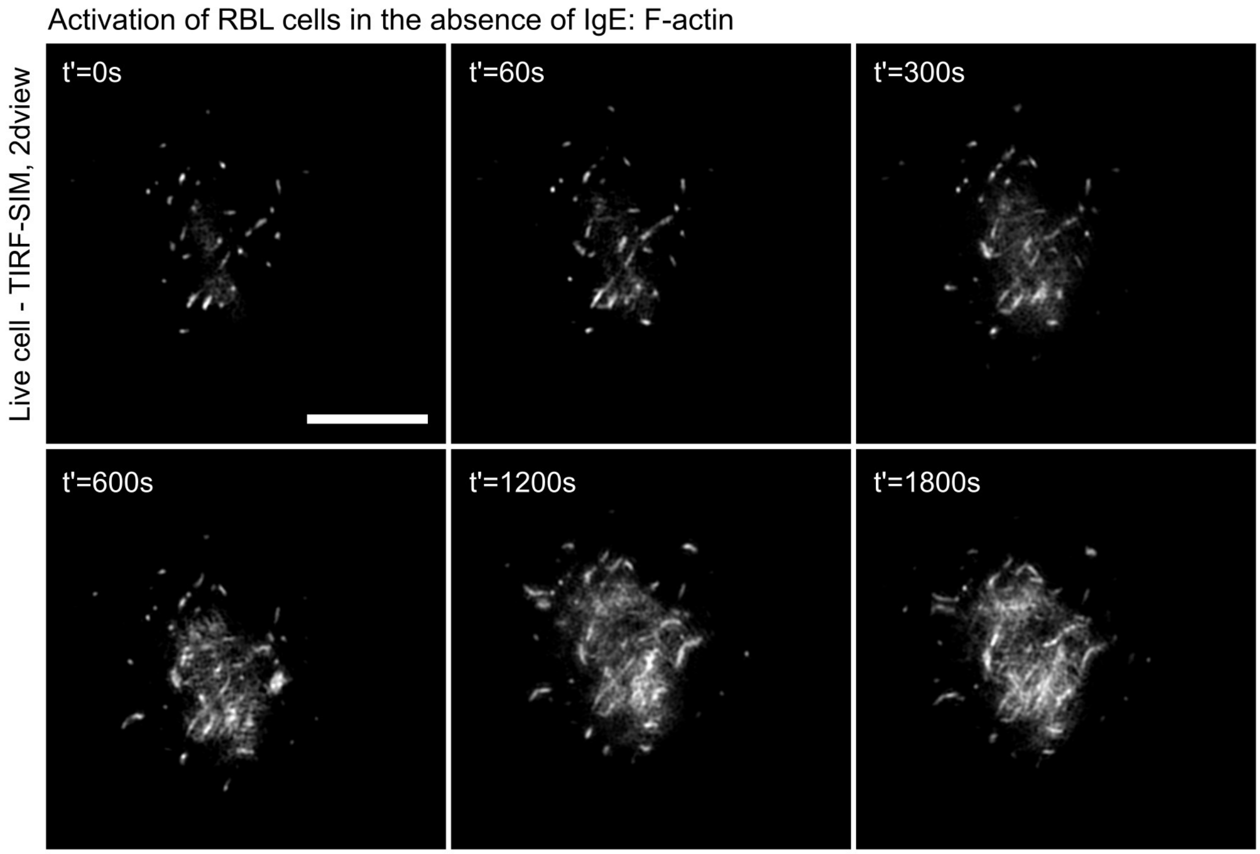

Supplementary Figure 3: Representative TIRF-SIM images of the F-actin network (grey, Lifeact-citrine) in live RBL cells in the absence of IgE specific activation. RBL cells did not undergo any of the stages (1-4) during their activation as observed during IgE mediated activation. Scale bar: 5µm.

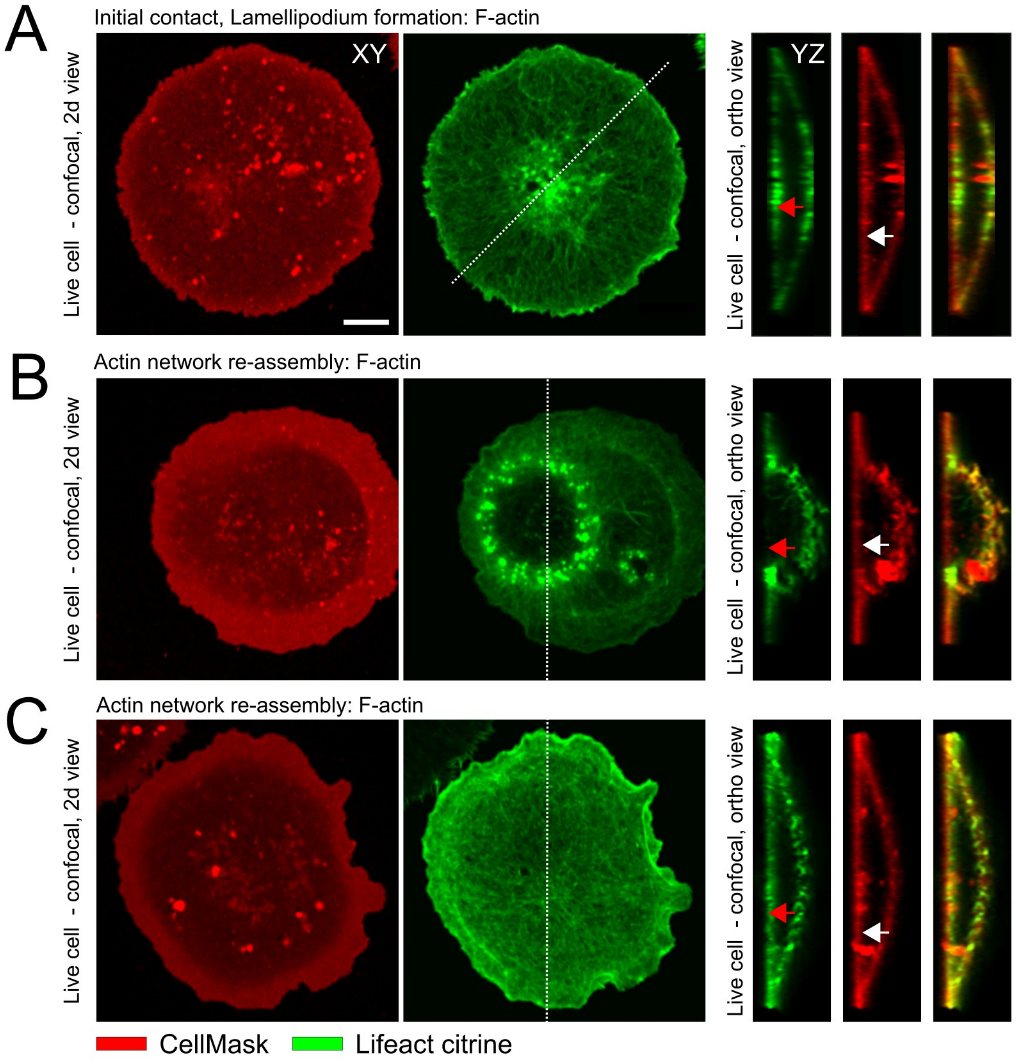

Supplementary Figure 4: Representative confocal images of the plasma membrane (red, Cell Mask) and F-actin (green, Lifeact-citrine) in live RBL cells during the different stages of activation: XY 2D-images of the basal interface (left) and images of the central axial YZ plane (right). (A) Initial contact: Cortical actin is localized to the basal membrane (red and white arrows). (B) Symmetry break during actin disassembly and re-assembly: Cortical actin is depleted at the basal membrane in the central dis-assembled area (red and white arrows). (C) After actin re-assembly: Cortical actin is again fully localized to the basal membrane (red and white arrows). Scale bar: 5µm.

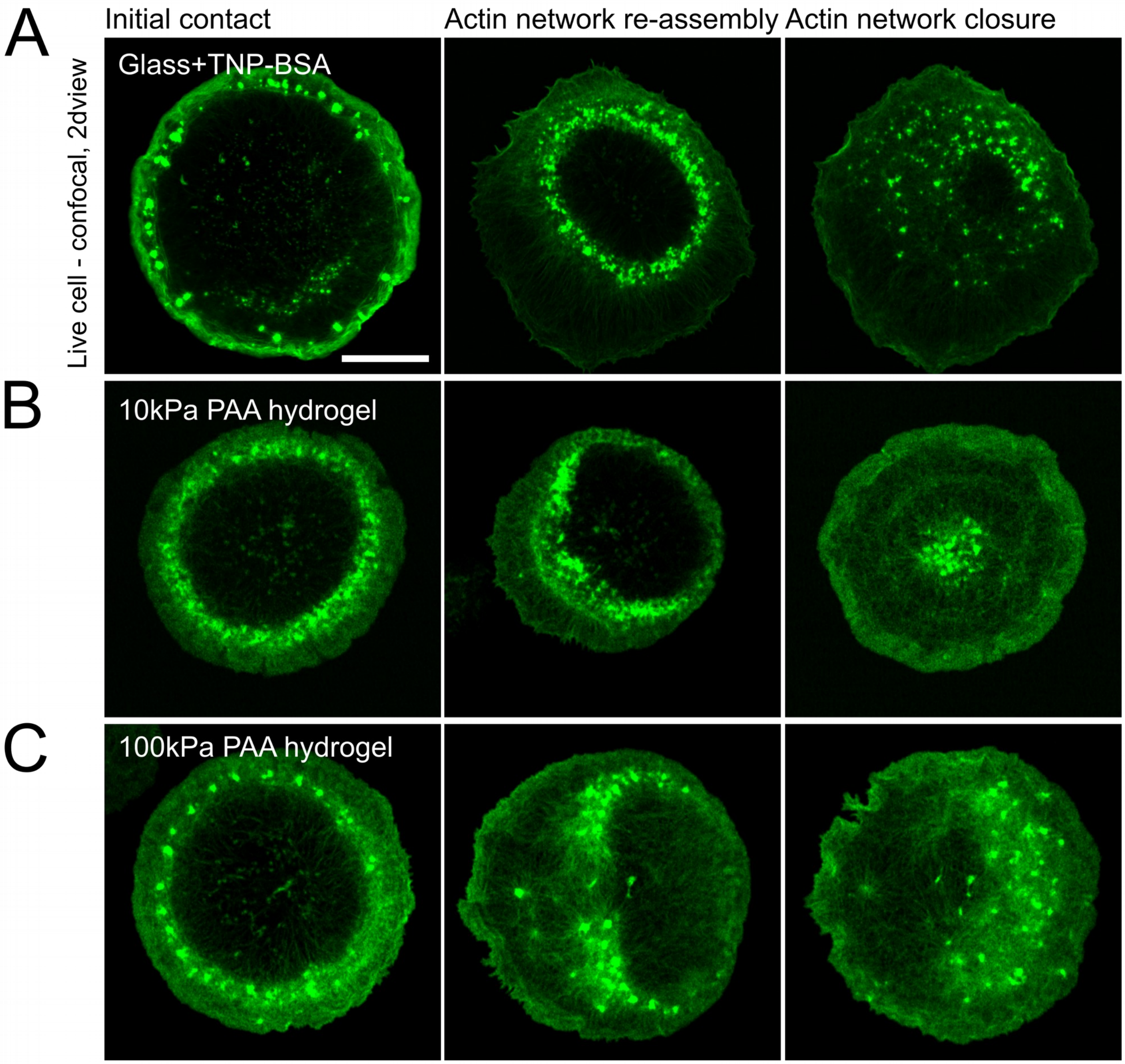

Supplementary Figure 5: Representative Airyscan confocal images of the F-actin network (green, Lifeact) at the different stages of activating live RBL cells on (A) TNP-BSA coated glass, (B) 10kPa and (C) 100kPa polyacrylamide hydrogel. Scale bar: 5µm.

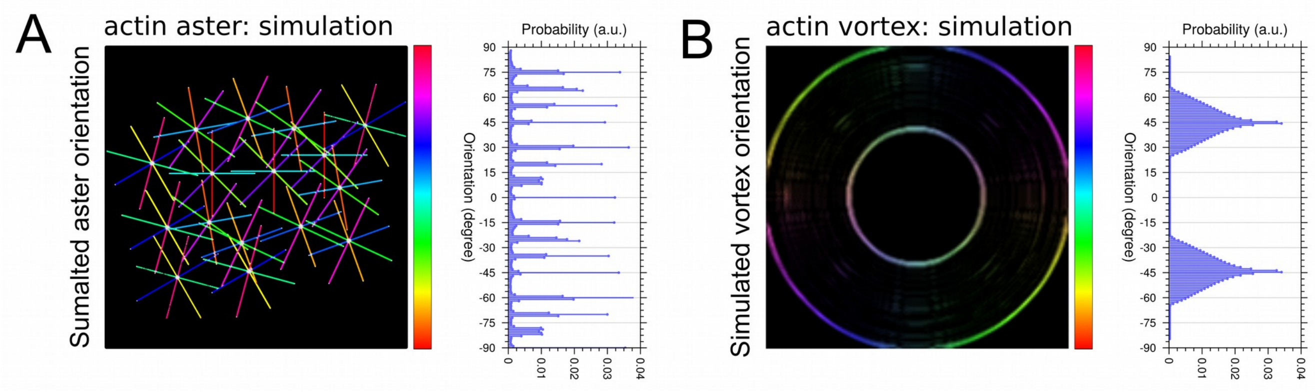

Supplementary Figure 6: Simulations of self-organizing actin patterns as proposed from in vitro data: asters (A) and vortices (B). Left panels: principal spatial organization of actin with color scale highlighting orientation of F-actin arms in space (light blue horizontal to red vertical, -90 to 90). Right panels: Frequency histogram of spatial orientations of F-actin arms (given as probability distribution), highlighting characteristic distributions for each case.

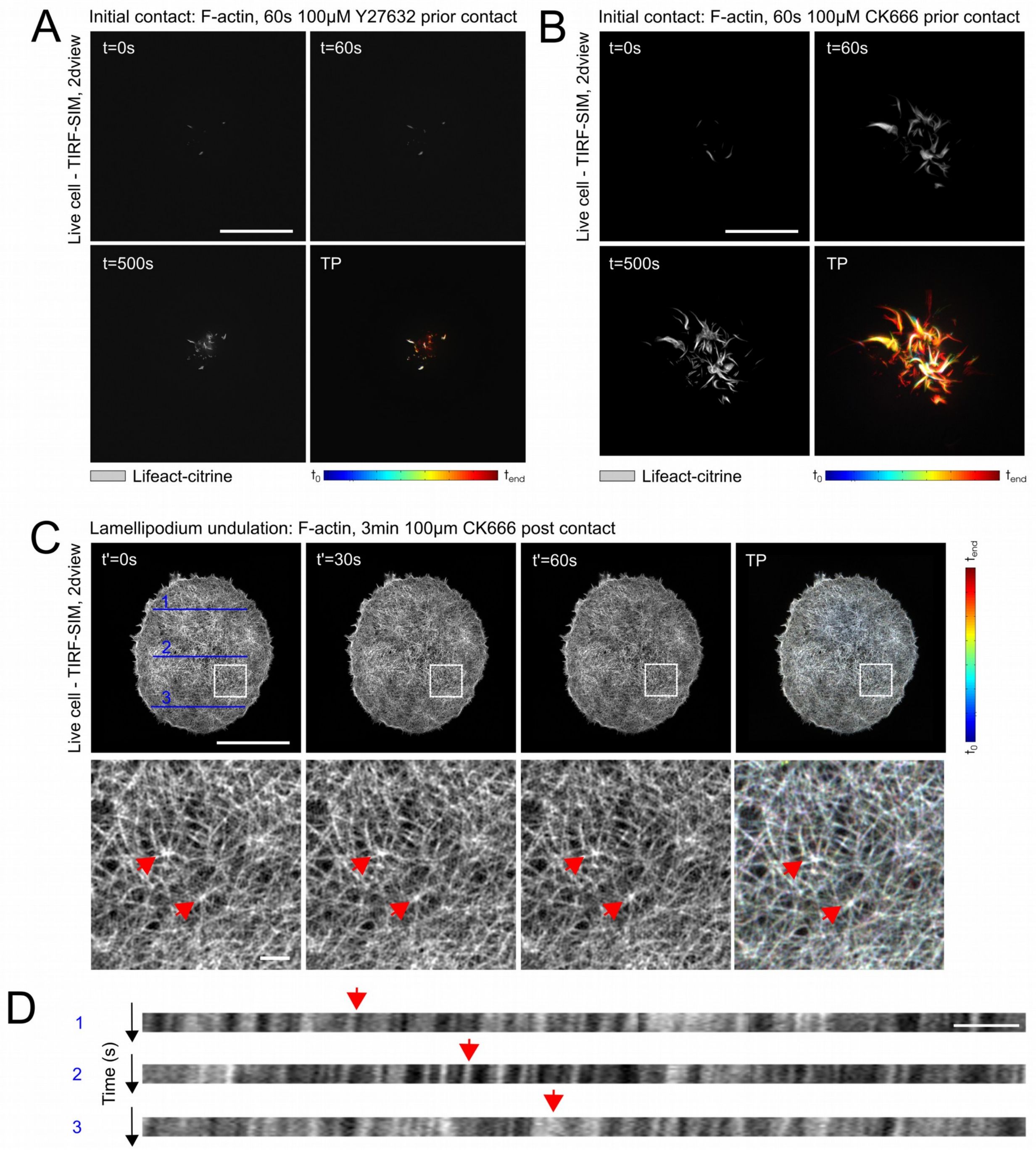

A
Initial contact: F-actin, 60s 100µM Y27632 prior contact
Live cell - TIRF-SIM, 2dview
t=0s
t=60s
t=500s
TP
Lifeact-citrine
t0
tend
B
Initial contact: F-actin, 60s 100µM CK666 prior contact
Live cell - TIRF-SIM, 2dview
t=0s
t=60s
t=500s
TP
Lifeact-citrine
t0
tend
C
Lamellipodium undulation: F-actin, 3min 100µm CK666 post contact
Live cell - TIRF-SIM, 2dview
t'=0s
t'=30s
t'=60s
TP
1
2
3
tend
t0
D
1
2
3
Time (s)

Supplementary Figure 7: Representative eTIRF-SIM experiments of the spatio-temporal dynamics of the actin network and patterns (LifeAct®-citrine) at the basal membrane during RBL cell activation following treatment by myosin II-inhibiting (Y27632) and Arp2/3-inhibiting (CK666) drugs. (A,B) Images taken at different times t after contact to the cover glass for Y27632 (A) and CK666 (B) treatment prior to contact formation, highlighting ceased contact formation and discontinued subsequent actin rearrangements. Scale bar: 10µm. (C) Images taken at different times t' starting at t=180s after contact formation and with addition of CK666, overviews (upper, scale bar: 10µm) and zoom-ins into white boxes (lower, scale bar: 10µm), highlighting an isotropically distributed cortical network dominated by asters (red arrows). (D) Temporal kymographs along the blue lines marked in C, highlighting the general arrest of actin network dynamics (red arrows, scale bar: 1µm).

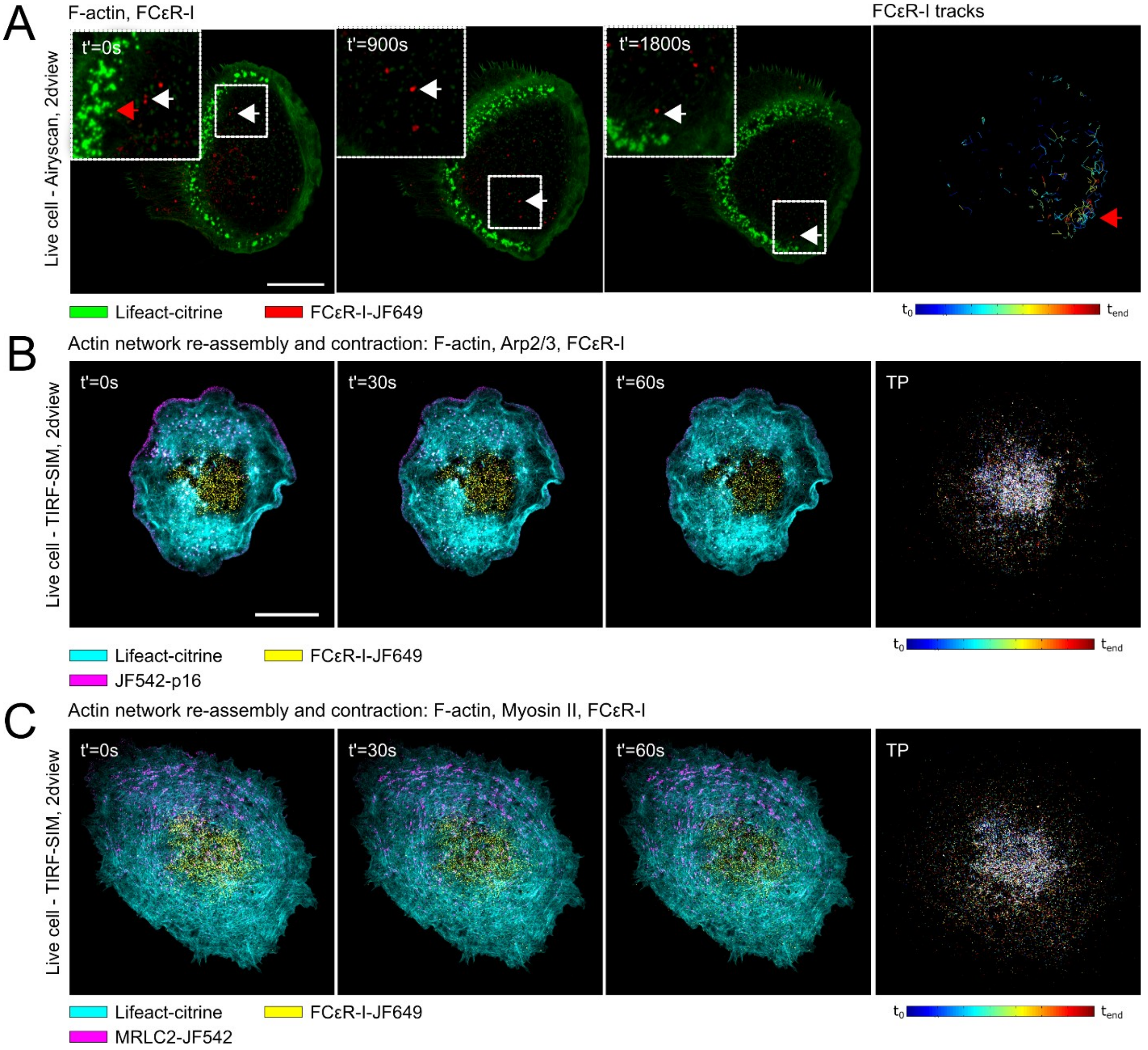

Supplementary Figure 8: (A) Representative Airy-scan images (overviews and zoom-ins into marked areas) of the cortical F-actin network (green, Lifeact citrine) and FCεR clusters (red, JF546) at different time points t' during actin re-assembly and (lower right) temporally coded tracks of FCεR clusters during this time period, demonstrating how the FCεR clusters (white arrows) moved ahead of the F-actin wave at comparable speed of network re-assembly (p=0.98, $v_{FC\ R}$ = 101±40 nm/s and v_{re} = 115 ±35 nm/s) and how the tracks of the clusters finished at the location of full actin network closure (red arrow). Scale bar: 5μm. (B-C) Images taken at different times t' during network re-assembly (starting at t≈1800s): (B) F-actin (cyan), Arp2/3 (magenta), and FCεR (yellow); (C) F-actin (cyan), myosin II (magenta), and FCεR (yellow). FCεR clusters propagated towards the center ahead of the re-assembling actin wave, where they remained immobile. Scale bar: 1μm.
